# Structure of an H3N2 influenza virus nucleoprotein

**DOI:** 10.1107/S2053230X2100635X

**Published:** 2021-06-29

**Authors:** Michael L. Knight, Haitian Fan, David L. V. Bauer, Jonathan M. Grimes, Ervin Fodor, Jeremy R. Keown

**Affiliations:** a Sir William Dunn School of Pathology, University of Oxford, South Parks Road, Oxford OX1 3RE, United Kingdom; b RNA Virus Replication Laboratory, Francis Crick Institute, Midland Road, London NW1 1AT, United Kingdom; c Division of Structural Biology, Welcome Centre for Human Genetics, University of Oxford, Roosevelt Drive, Oxford OX3 7BN, United Kingdom

**Keywords:** influenza, H3N2 influenza virus nucleoprotein, X-ray crystallography, nucleoprotein, RNA-binding protein

## Abstract

Influenza A viruses of the H1N1 and H3N2 subtypes are responsible for seasonal epidemic events. The influenza nucleoprotein (NP) binds to the viral genomic RNA and is essential for its replication. Efforts are under way to produce therapeutics and vaccines targeting the NP. Despite this, no structure of an NP from an H3N2 virus has previously been determined. Here, the structure of the A/Northern Territory/60/1968 (H3N2) influenza virus NP is presented at 2.2 Å resolution. The structure is highly similar to those of the A/WSN/1933 (H1N1) and A/Hong Kong/483/97 (H5N1) NPs. Nonconserved amino acids are widely dispersed both at the sequence and structural levels. A movement of the 73–90 RNA-binding loop is observed to be the key difference between the structure determined here and previous structures. The data presented here increase the understanding of structural conservation amongst influenza NPs and may aid in the design of universal interventions against influenza.

## Abstract 2

The influenza virus nucleoprotein binds to the viral RNA genome and is essential for virus replication. Here, the structure of the nucleoprotein from an H3N2 virus is presented at 2.2 Å resolution.

## 1. Introduction   

Influenza A viruses (IAVs) make a large contribution to the seasonal influenza burden and have established pandemic potential. The major antigenic components of IAVs are the hemagglutinin and neuraminidase proteins that decorate the viral envelope. These proteins are used to classify IAVs into different subgroups by assigning them H and N numbers (for example H1N1, H3N2, H5N1 *etc.*). IAVs have a broad host range, covering a wide variety of mammals and birds. However, currently only IAVs of two subtypes, H1N1 and H3N2, exhibit sustained human-to-human transmission.

The IAV genome consists of eight segments of negative-sense RNA (vRNA), each encoding at least one essential protein. Each segment is assembled into a ribonucleoprotein complex, with the 5′ and 3′ termini both bound by the trimeric influenza virus polymerase. The rest of the segment is bound, on average, every 25 nucleobases (Ortega *et al.*, 2000 ▸; Hutchinson *et al.*, 2014 ▸) by the 56 kDa influenza virus nucleoprotein (NP). The NP forms homo-oligomers along the vRNA by inserting a loop, located close to its C-terminal tail, into the body domain of a neighbouring NP. NP is a multifunctional protein that influences the structure of the vRNA (Lee *et al.*, 2017 ▸; Williams, Townsend *et al.*, 2018 ▸; Dadonaite *et al.*, 2019 ▸), with essential roles in nuclear trafficking of vRNAs (O’Neill *et al.*, 1995 ▸) and replication (Portela & Digard, 2002 ▸).

Structures have been determined of NPs from influenza A, B (Ng *et al.*, 2012 ▸) and D (Donchet *et al.*, 2019 ▸) viruses. For IAVs, these include the A/WSN/1933 H1N1 (WSN; Ye *et al.*, 2006 ▸) and A/Hong Kong/483/97 H5N1 (HK97; Ng *et al.*, 2008 ▸) viruses. The structure of a monomeric mutant of the WSN NP, containing an R416A mutation (located in the oligomerization loop), has also been determined (Chenavas *et al.*, 2013 ▸). No high-resolution structure has been determined of an NP associated with RNA. However, mutational analysis and structural information suggest that RNA binding is mediated by a basic groove located between the head and body domains (Ye *et al.*, 2006 ▸; Elton *et al.*, 1999 ▸). This basic groove is thought to associate with the phosphate backbone of the RNA. The interaction does not exhibit sequence specificity (Williams, Townsend *et al.*, 2018 ▸), and NP also associates with single-stranded DNA (Newcomb *et al.*, 2009 ▸).

Efforts are under way to develop antiviral therapeutics targeting NP (Hu *et al.*, 2017 ▸), as well as to use it as a target for universal vaccines that are effective against multiple strains of influenza (Sun *et al.*, 2020 ▸; Pleguezuelos *et al.*, 2020 ▸). This work may be aided by a greater understanding of NP conservation at a structural level; however, no structure has previously been determined of an NP from an H3N2 virus. Here, we present the structure of the NP from the A/Northern Territory/60/1968 (H3N2) influenza virus (NT60) and discuss how it differs from previously determined influenza virus NP structures.

## 2. Materials and methods   

### 2.1. Macromolecule production   

The sequence for the NT60 NP containing an R416A mutation was amplified from the vector pFL-TAP-NP R416A (Turrell, 2015 ▸). The R416A mutation was used as it has previously been shown to make the NP monomeric, and we reasoned that this construct would be more suitable for crystallization (Ye *et al.*, 2006 ▸). This sequence was optimized for expression in *Spodoptera frugiperda* insect cells. Primers containing overhangs were used to generate a fragment with BamHI and EcoRI restriction sites at either end. The resulting fragment was ligated into the pGEX-6P-1 expression vector (for expression with an N-terminal glutathione *S*-transferase tag) and transformed into competent DH5α cells. Plasmid DNA was then extracted using a QIAprep Spin Miniprep kit (Qiagen) and successful integration of the insert was confirmed by sequencing. The constructs were transformed into *Escherichia coli* BL21 (DE3) cells and a 50%(*v*/*v*) glycerol stock was produced and stored at −80°C.

Bacteria from the glycerol stock were used to inoculate 10 ml lysogeny broth (LB) containing ampicillin (100 µg ml^−1^) and grown at 37°C overnight. The following day, the overnight culture was used to inoculate 2 l LB medium at a ratio of 1:100. Once an OD_600_ of 0.6 had been reached, protein expression was induced by the addition of isopropyl β-d-1-thiogalactopyranoside to a final concentration of 1 m*M* and the temperature was reduced to 18°C. After 16 h, the bacteria were pelleted by centrifugation at 4000*g* for 15 min at 4°C. The pellets were then resuspended in 25 ml wash buffer [50 m*M* HEPES–NaOH pH 7.5, 500 m*M* NaCl, 10%(*v*/*v*) glycerol, 0.05%(*w*/*v*) octyl β-d-1-thioglucopyranoside (OTG)] with the addition of 50 µl 1 *M* DTT, 2.5 mg RNase A, one SIGMAFAST protease inhibitor tablet (Sigma), 10 µl Benzonase Nuclease (Sigma) and 35 mg lysozyme. The pellet was then resuspended prior to lysis by sonication. The lysed cells were centrifuged at 35 000*g* for 45 min at 4°C. 1 ml Glutathione Sepharose 4B beads (GE Healthcare) was added to the clarified supernatant, which was incubated at 4°C for 3 h with gentle rotation. The beads were collected by centrifugation at 2000*g* at 4°C for 3 min and the supernatant was removed. The beads were then washed five times with high-salt wash buffer [50 m*M* HEPES–NaOH pH 7.5, 1.5 *M* NaCl, 10%(*v*/*v*) glycerol, 0.05%(*w*/*v*) OTG]. The beads were washed again with wash buffer containing 5 m*M* DTT before being resuspended in 10 ml wash buffer supplemented with 5 m*M* DTT, 0.2 mg HRV 3C protease and 5 µl Benzonase Nuclease. After incubation overnight at 4°C with gentle rotation, the beads were pelleted at 2000*g* for 5 min at 4°C. The supernatant containing released protein was then collected and concentrated.

The concentrated protein was applied onto a Superdex 200 Increase 10/300 GL column (GE Healthcare) equilibrated with a buffer consisting of 25 m*M* HEPES–NaOH pH 7.5, 150 m*M* NaCl. The fractions containing the NP were pooled and concentrated in a 30 kDa Millipore Protein Concentrator to a protein concentration of ∼20 mg ml^−1^. The correct molecular weight of the protein was confirmed by SDS–PAGE with Coomassie Blue staining. The protein was flash-frozen using liquid nitrogen and stored at −80°C. Macromolecule-production information is summarized in Table 1 ▸.

### 2.2. Assessment of nucleic acid binding   

The ability of the purified R416A NP to bind RNA was assessed by mixing a 1:1 molar ratio of NP with RNA of either five (5′-AGUAG-3′) or 14 (5′-CCUCUGCUUCUGCU-3′) nucleotides in length in buffer consisting of 25 m*M* HEPES–NaOH pH 7.5, 150 m*M* NaCl. After incubation for 10 min at room temperature, the mixture was subjected to size-exclusion chromatography (SEC) as described earlier. The NP-containing fraction was collected and the *A*_260_/*A*_280_ ratio was assessed. The ability of the purified NP to bind DNA was assessed by mixing the purified NP in a 4:1 molar ratio with a 100-nucleotide DNA in buffer consisting of 25 m*M* HEPES–Na pH 7.5, 150 m*M* NaCl. After incubation for 10 min at room temperature, the mixture was subjected to SEC.

RNA binding was further investigated using a ThermoFluor assay (Walter *et al.*, 2012 ▸) with a G nucleotide, a 5′-AG-3′ dinucleotide or the oligonucleotides 5′-UAUGAGGC-3′, 5′-AAAAAAAAAAAA-3′ and 5′-GUAUAUGAGGCCCA-3′. Each sample was analysed in triplicate in a 96-well PCR plate in an Mx3005P qPCR System (Agilent). The excitation filter was set to 492 nm and the emission filter to 585 nm. Data were collected in the range 25–95°C using an ‘expanding sawtooth’ profile in which fluorescence is always recorded at 25°C after 30 s incubations at increasing temperatures. A total volume of 40 µl was used (buffer: 25 m*M* HEPES–NaOH pH 7.5, 150 m*M* NaCl) containing 3 µg NP, 20 µ*M* RNA and a 1:100 dilution of SYPRO Orange (Invitrogen). Melting curves were fitted and melting temperatures were determined using the *JTSA* web server (Bond, 2017 ▸).

### 2.3. Crystallization   

The protein was diluted to 10 mg ml^−1^ in a buffer consisting of 25 m*M* HEPES–NaOH pH 7.5, 150 m*M* NaCl. Crystallization trials were undertaken in Swissci 3-drop plates with a drop volume of 200 nl. The conditions which yielded the best diffracting crystal are summarized in Table 2 ▸.

### 2.4. Data collection and processing   

A number of data sets were collected from cryocooled crystals at Diamond Light Source (DLS), Didcot, UK. Data-collection parameters and merging statistics for the best-diffracting crystal are summarized in Table 3 ▸. Data were processed using *autoPROC* (Vonrhein *et al.*, 2011 ▸) and an anisotropic cutoff was applied to the data using *STARANISO* (Tickle *et al.*, 2018 ▸). The data were weakly anisotropic and were thus truncated anisotropically, giving rise to low spheri­cal completeness and *I*/σ(*I*) values.

### 2.5. Structure solution and refinement   

The data quality was assessed for pathologies using *phenix.xtriage* (Zwart *et al.*, 2005 ▸). The structure was then solved by molecular replacement in *Phaser* (McCoy *et al.*, 2007 ▸) using a previously determined WSN R416A NP model (PDB entry 3zdp; Chenavas *et al.*, 2013 ▸). Iterative rounds of automated refinement were performed in *phenix.refine* (Afonine *et al.*, 2012 ▸) and manual model adjustment in *Coot* (Emsley *et al.*, 2010 ▸). *MolProbity* (Williams, Headd *et al.*, 2018 ▸) was used throughout for model validation. Data have been deposited in the PDB with the accession code 7nt8. Structural figures were all prepared using *ChimeraX* (Pettersen *et al.*, 2021 ▸). Refinement statistics are summarized in Table 4 ▸.

## 3. Results and discussion   

**Figure 1:**
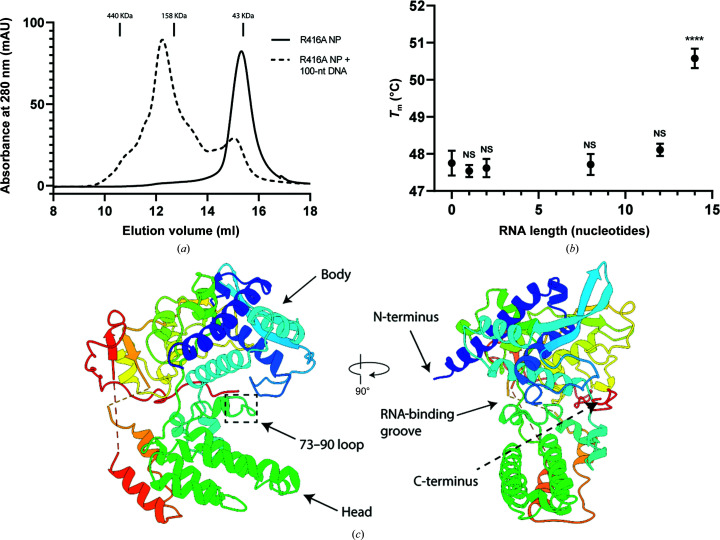
(*a*) The absorbance at 280 nm from SEC of the NT60 R416A NP either in the presence or absence of a 100-nucleotide DNA. (*b*) The melting temperature of the NT60 R416A NP in the presence of different lengths of nucleic acids. (*c*) The structure of the NT60 R416A NP in ribbon representation.

The NT60 monomeric mutant R416A NP was expressed in *E. coli*. After multiple high-salt washes and nuclease treatment, the protein was purified by SEC. A single symmetric peak was observed during SEC, which eluted at a volume consistent with the mass of monomeric NP (Fig. 1 ▸ *a*). The peak position and the *A*_260_/*A*_280_ ratio of 0.49 indicate that the NP was successfully stripped of endogenous nucleic acids from the expression host.

The ability of the monomeric R416A NP to bind RNA was investigated using a ThermoFluor assay, in which the melting temperature of the NP was determined in association with different length RNAs (Fig. 1 ▸ *b*). The melting temperature of the NP mixed with a 14-nucleotide RNA was increased by 2.8°C compared with that of the NP in the absence of RNA (*p* < 0.0001, one-way ANOVA), suggesting that this association increased the stability of the NP. Shorter oligo­ribonucleotides did not significantly increase the melting temperature, although it cannot be excluded that the NP could be stabilized by shorter length RNAs with different sequences.

RNA binding was further assessed by mixing the purified R416A NP in a 1:1 molar ratio with a five- or 14-nucleotide RNA. The *A*_260_/*A*_280_ ratio of the NP-containing fraction was then measured post-SEC. The NP mixed with the five-nucleotide RNA gave an *A*_260_/*A*_280_ value of 0.53 and the NP mixed with the 14-nucleotide RNA gave a value of 1.05. This indicates that the 14-nucleotide RNA is able to associate with the NP strongly enough to remain bound through SEC, but the five-nucleotide RNA is not. The ability of the R416A NP to bind DNA was also assessed by mixing purified NP in a 4:1 molar ratio with a 100-nucleotide DNA and performing SEC. This produced a second, earlier elution peak (Fig. 1 ▸ *a*) that is likely to represent multiple NPs associating with a single piece of DNA (100-nucleotide DNA has a mass of ∼30.7 kDa).

A range of crystallization trials were set up for the NT60 R416A NP both in the presence or absence of a 1.7-fold molar excess of 14-nucleotide RNA. Despite its ability to bind to and be stabilized by 14-nucleotide RNA, no RNA could be resolved from the crystals produced in its presence. The best-diffracting crystal produced in the absence of RNA gave a maximum resolution of 2.2 Å (Fig. 1 ▸ *c*), with two NPs per asymmetric unit (referred to as chains *A* and *B*), in space group *P*2_1_. The NP structure consists of head and body domains composed primarily of α-helices. A basic groove, thought to be the site of RNA binding, lies at the interface of these two domains. This groove contains a large number of arginine and lysine residues that, whilst located closely to­gether in the folded structure, are dispersed widely in the protein sequence. Both NP chains are resolved from residues 21 to 389. Most of the oligomerization loop could not be resolved, with residues 390–417 and 390–437 disordered in chains *A* and *B*, respectively. At the C-terminus, residues 452–461 and 497–498 were not resolved.

**Figure 2:**
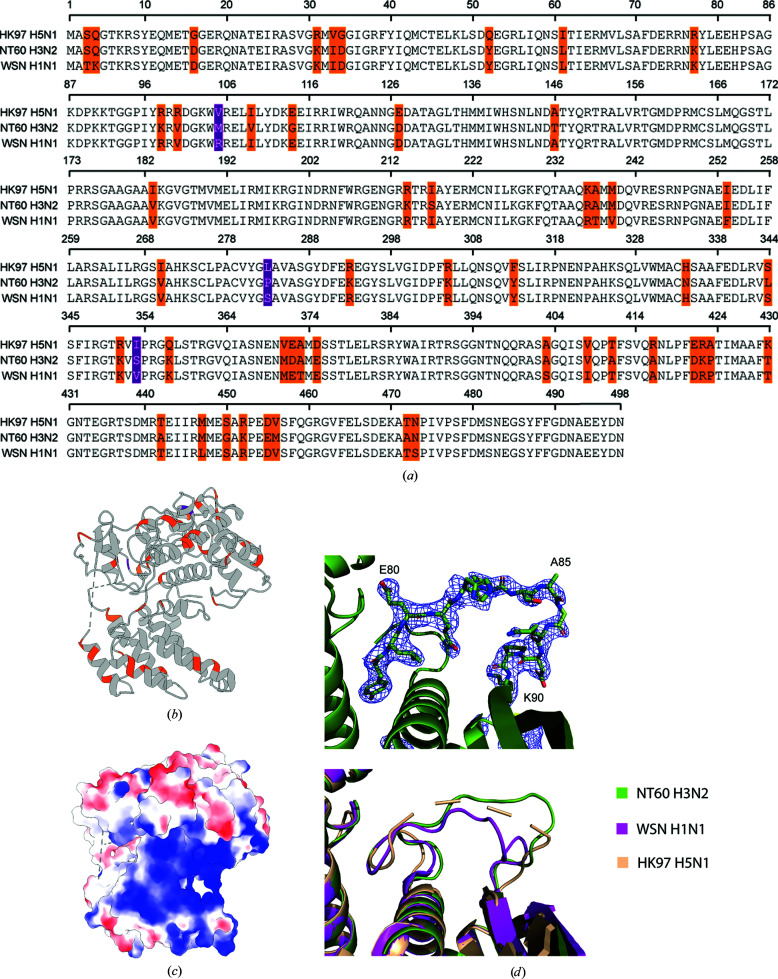
(*a*) Sequence alignment of the NT60 (H3N2), WSN (H1N1) and HK97 (H5N1) NP sequences. (*b*) Ribbon representation of the NT60 R416A NP showing amino-acid sequence conservation between the NT60, WSN and HK97 NPs (grey, conserved; orange, one sequence differs; purple, all three sequences differ). (*c*) Surface representation of the NT60 R416A NP showing the surface-charge distribution (blue, basic; red, acidic). (*d*) Top: the electron density (level 1.0) of residues 78–90 in the NT60 R416A H3N2 structure. Bottom: a comparison to the positioning of the 73–90 loop in the WSN R416A and HK97 NP structures.

The amino-acid sequence of the NP is highly conserved amongst IAVs. The NT60 NP shares 93.6% and 91.4% amino-acid sequence identity with the WSN (H1N1) and HK97 (H5N1) NPs, respectively, for which structures have previously been determined. The structure of the NT60 R416A NP is highly similar to other published IAV NP structures, with root-mean-square deviations of 1.2 Å compared with the WSN R416A NP (across 439 pairs), 4.0 Å compared with the WSN NP (across 393 pairs) and 5.5 Å compared with the HK97 NP (across 429 pairs). The differences in the amino-acid sequences of these three IAV NPs are widely dispersed both at the sequence level (Fig. 2 ▸ *a*) and the structural level (Fig. 2 ▸ *b*). Only one nonconserved residue is present in the basic region forming the predicted RNA-binding groove (Fig. 2 ▸ *c*). A lysine at position 77 in the NT60 and WSN NPs is replaced by an arginine in the HK97 NP, maintaining the basic charge.

The major difference between the structure presented here and those previously determined is the position of the 73–90 loop, the deletion of which produces an approximately fivefold decrease in RNA-binding affinity (Ng *et al.*, 2008 ▸). In the H1N1 R416A structure, residues 82–89 of this loop extend into the putative RNA-binding site, whilst in the H5N1 model these residues are disordered and were not modelled. In chain *A* of our model we observe that residues 82–89 point away from the RNA-binding groove (Fig. 2 ▸ *d*). The density for this region is incomplete in chain *B*. The 73–81 region of the loop appears to adopt a more conserved structure. This region of the loop appears to be critical to RNA binding, with simultaneous mutation of the Arg74 and Arg75 residues along with Arg174, Arg175 and Arg221 (which are located on the opposite side of the RNA-binding groove) having been shown to abolish RNA binding (Ng *et al.*, 2008 ▸).

We observe that the C-terminus of the NT60 R416A NP folds towards the RNA-binding groove. This was observed for the R416A WSN monomeric mutant NP structure but not in the oligomeric structures. It has been suggested that this folding of the tail reduces the positive charge of this groove (Chenavas *et al.*, 2013 ▸) and may explain the reduced RNA-binding affinity of the monomeric mutant (Elton *et al.*, 1999 ▸).

We have presented the structure of the NT60 R416A NP at 2.2 Å resolution. The structure is highly similar to that of previously reported NP structures, but contributes to our understanding of structural conservation amongst the NPs from IAVs. This may aid in the design of therapeutics with activity against multiple subtypes of IAV to improve responses to future epidemic and pandemic events.

## Supplementary Material

PDB reference: H3N2 influenza virus nucleoprotein, 7nt8

## Figures and Tables

**Table 1:** Macromolecule-production information

Source organism	A/Northern Territory/60/1968 (H3N2) influenza virus
DNA source	pFL-TAP-NP R416A
Forward primer†	GGATCCATGGCTTCCCAGGGTAC
Reverse primer‡	GAATTCTTAGTTGTCGTATTCCTCAGC
Expression vector	pGEX-6P-1
Expression host	*E. coli* BL21 (DE3) cells
Complete amino-acid sequence of the construct produced§	GPLGSMASQGTKRSYEQMETDGERQNATEIRASVGKMIDGIGRFYIQMCTELKLSDYEGRLIQNSLTIERMVLSAFDERRNKYLEEHPSAGKDPKKTGGPIYKRVDGKWMRELVLYDKGEIRRIWRQANNGDDATAGLTHMMIWHSNLNDTTYQRTRALVRTGMDPRMCSLMQGSTLPRRSGAAGAAVKGVGTMVMELIRMIKRGINDRNFWRGENGRKTRSAYERMCNILKGKFQTAAQRAMMDQVRESRNPGNAEIEDLIFLARSALILRGSVAHKSCLPACVYGPAVASGYDFEKEGYSLVGIDPFKLLQNSQVYSLIRPNENPAHKSQLVWMACNSAAFEDLRVLSFIRGTKVSPRGKLSTRGVQIASNENMDAMESSTLELRSRYWAIRTRSGGNTNQQRASAGQISVQPAFSVQANLPFDKPTIMAAFTGNTEGRTSDMRAEIIRMMEGAKPEEMSFQGRGVFELSDEKAANPIVPSFDMSNEGSYFFGDNAEEYDN

† The BamHI restriction site is underlined.

‡ The EcoRI restriction site is underlined.

§ Residues retained after cleavage that are not part of the NP sequence are underlined.

**Table 2:** Crystallization

Method	Vapour diffusion
Plate type	Swissci 3-drop
Temperature (K)	293
Protein concentration (mg ml^−1^)	10
Buffer composition of protein solution	25 m*M* HEPES–NaOH pH 7.5, 150 m*M* NaCl
Composition of reservoir solution	10%(*w*/*v*) PEG 8000, 20%(*v*/*v*) ethylene glycol, 0.02 *M* of each alcohol [0.2 *M* 1,6-hexanediol, 0.2 *M* 1-butanol, 0.2 *M* (*RS*)-1,2-propanediol, 0.2 *M* 2-propanol, 0.2 *M* 1,4-butanediol, 0.2 *M* 1,3-propanediol], 0.1 *M* MES/imidazole pH 6.5
Volume and ratio of drop	200 nl (1:1)
Volume of reservoir (µl)	30

**Table 3:** Data collection and processing Values in parentheses are for the outer shell.

Diffraction source	I24, DLS
Wavelength (Å)	0.9686
Temperature (K)	100
Detector	PILATUS 6M, Dectris
Space group	*P*2_1_
*a*, *b*, *c* (Å)	87.78, 63.38, 105.95
α, β, γ (°)	90.0, 98.3, 90.0
Resolution range (Å)	86.85–2.22 (2.30–2.22)
Total No. of reflections	250461 (11323)
No. of unique reflections	37998 (1900)
Completeness (%) (ellipsoidal)	90.9 (55.8)
Multiplicity	6.6 (6.0)
〈*I*/σ(*I*)〉	8.1 (1.6)
*R*_r.i.m._	0.07 (0.56)
Overall *B* factor from Wilson plot (Å^2^)	46.1

**Table 4:** Structure solution and refinement Values in parentheses are for the outer shell.

Resolution range (Å)	72.21–2.22 (2.30–2.22)
Completeness (%) (spherical)	66.6 (3.4)
No. of reflections, working set	38052 (203)
No. of reflections, test set	1830 (11)
Final *R*_cryst_	0.21 (0.26)
Final *R*_free_	0.26 (0.38)
No. of non-H atoms	
Total	6813
Protein	6762
Water	51
R.m.s. deviations	
Bonds (Å)	0.003
Angles (°)	0.55
Average *B* factors (Å^2^)	
Protein	60.0
Water	46.5
Ramachandran plot	
Most favoured (%)	97.16
Allowed (%)	2.73
*MolProbity* score	1.28
